# Association of serum uric acid with incident vascular calcification in patients with type 2 diabetes mellitus: a retrospective cohort study

**DOI:** 10.3389/fendo.2026.1893598

**Published:** 2026-07-28

**Authors:** Xinyu Li, Taoyuan He, Guosheng Li, Xue Liang, Bing Wang, Yudan Liu, Wei Zhao, Zhu Zhu, Xuhan Liu

**Affiliations:** 1 Department of Endocrinology, Central Hospital of Dalian University of Technology, Dalian, China; 2 Laboratory Pathology Department, Ningbo Clinical Pathology Diagnosis Center, Ningbo, China; 3 Department of Respiratory Medicine, Yanhu District People’s Hospital of Yuncheng, Yuncheng, China; 4 Department of Neuroendocrine Pharmacology, School of Pharmacy, China Medical University, Shenyang, China

**Keywords:** retrospective cohort study, risk stratification, serum uric acid, type 2 diabetes mellitus, vascular calcification

## Abstract

**Background:**

Vascular calcification (VC) is common among patients with type 2 diabetes mellitus (T2DM) and is associated with adverse cardiovascular outcomes. Although experimental evidence suggests that elevated serum uric acid (SUA) may promote VC, its clinical relevance in patients with T2DM remains unclear.

**Methods:**

We conducted a retrospective cohort study based on electronic medical records from the Central Hospital of Dalian University of Technology. The study included 665 hospitalized patients with T2DM who had no evidence of VC on baseline pulmonary computed tomography (CT) and underwent follow-up pulmonary CT. Incident VC was defined as newly detected, radiologically visible coronary artery calcification (CAC), aortic calcification, or both on mediastinal-window pulmonary CT. Associations between SUA and incident VC were assessed using Kaplan–Meier analysis, multivariable Cox regression, restricted cubic spline (RCS)analysis, subgroup analyses, and sensitivity analyses. Predictive performance was assessed using time-dependent receiver operating characteristic (ROC) analysis with inverse probability of censoring weighting (IPCW).

**Results:**

During a median follow-up of 2.25 years, incident VC developed in 315 patients. In the fully adjusted model, each 1-standard-deviation (SD) increase in SUA was associated with a higher risk of incident VC (HR, 1.271; 95% CI, 1.138–1.420; P < 0.001). Compared with the lowest SUA quartile, the third and fourth quartiles were associated with higher risks of incident VC (HR, 1.634; 95% CI, 1.125–2.375; P = 0.010; and HR, 2.075; 95% CI, 1.432–3.006; P < 0.001, respectively). RCS analysis showed an approximately linear association between SUA and incident VC. Time-dependent areas under the curve (AUCs) for SUA were 0.666, 0.730, 0.777, and 0.847 at 1, 3, 5, and 10 years, respectively. Adding SUA to the fully adjusted base model yielded modest improvements in discrimination and prediction error.

**Conclusion:**

Among patients with T2DM, higher SUA levels were associated with an increased risk of incident, radiologically visible macrovascular calcification. SUA may help identify patients at higher risk of VC; however, the data-derived threshold of approximately 300 μmol/L should be considered exploratory and requires external validation. Whether lowering SUA reduces the risk of VC remains to be determined in prospective interventional studies.

## 1 Introduction

Vascular calcification (VC) is the abnormal deposition of calcium phosphate, primarily hydroxyapatite, within the vascular walls. It serves as a common pathophysiological mechanism underlying several diseases, including atherosclerosis, diabetes mellitus (DM), chronic kidney disease (CKD), and hypertension. VC is a major contributor to the high incidence and mortality rates of cardiovascular and cerebrovascular diseases (1). DM is a systemic metabolic disorder resulting from the combined effects of genetic and environmental factors, characterized primarily by abnormally elevated blood glucose levels. This condition substantially increases the risk of complications, impairs quality of life, and increases mortality (2). Existing clinical studies provide substantial evidence supporting a clear association between DM and VC (3–6). Mechanistically, mounting evidence suggests that several factors associated with metabolic syndrome (MetS) and linked to type 2 DM (T2DM) may promote VC. These factors include oxidative stress, endothelial dysfunction, dyslipidemia, mineral metabolism disturbances, and elevated synthesis of pro-inflammatory cytokines (7). Recent studies suggest that under high glucose (HG) conditions induced by DM, disrupted extracellular pyrophosphate (PPi) metabolism, along with the action of extracellular vesicles via the circ_0008362/miR-1251-5p/Runx2 axis, promotes VC (8, 9).

Uric acid (UA) is the end product of purine metabolism. At physiological concentrations, serum uric acid (SUA) plays a key role in combating oxidative stress, protecting against DNA damage, and scavenging free radicals (10, 11). However, excessively high SUA levels promote oxidative reactions that trigger vascular smooth muscle proliferation, endothelial dysfunction, and local tissue damage, contributing to conditions such as gout and cardiovascular disease (12). Excessively low SUA levels may contribute to the development of conditions such as Alzheimer’s and Parkinson’s diseases (13–15). The current pooled prevalence of hyperuricemia in China is 16.4%, demonstrating a significant upward trend (16). Basic research using animal and cellular models suggests that HUA inhibits osteoblast differentiation and proliferation both *in vivo* and *in vitro*, while promoting the osteoblast-like transdifferentiation of vascular smooth muscle cells (VSMCs), thereby increasing the risk of VC (17). However, existing clinical evidence is mixed, and the clinical relevance of SUA levels to VC has not been conclusively established (18–20). In patients with T2DM, the association between SUA levels and VC also remains.

In summary, to further explore the relationship between SUA and VC in patients with T2DM, this study conducted a follow-up investigation of hospitalized T2DM patients at the Central hospital of Dalian University of Technology from January 2010 to January 2023. The aim was to determine whether SUA is an independent risk factor for the development of VC.

## 2 Methods

### 2.1 Study population and grouping

This retrospective cohort study initially enrolled 1,557 patients with T2DM, aged 28 to 92 years, who were hospitalized at least twice at the Central Hospital of Dalian University of Technology between January 2010 and January 2023. The data for this study were extracted from the routine clinical records of hospitalized patients. Since all patients provided general informed consent upon admission, and the study was approved by the Ethics Committee of Central Hospital of Dalian University of Technology, the need for additional informed consent was waived.

Inclusion Criteria: Diagnosed with T2DM; age ≥18 years; at least two pulmonary CT examinations; no VC detected on the initial admission pulmonary CT; biochemical testing during the initial hospitalization, including serum uric acid levels; complete clinical data. Exclusion Criteria: Pregnancy or lactation; malignant tumors; genetic disorders; neurological diseases (e.g., traumatic brain injury or epilepsy); severe psychiatric disorders (e.g., schizophrenia); acute or chronic infections; acute complications; concomitant hematologic disorders, severe heart failure, liver failure, renal failure, or other serious conditions; use of UA synthesis inhibitors, uricosuric agents, diuretics, or other drugs affecting UA metabolism within the past 12 months; concomitant disorders affecting calcium-phosphorus metabolism, such as parathyroid dysfunction; missing clinical information.

**Figure 1:**
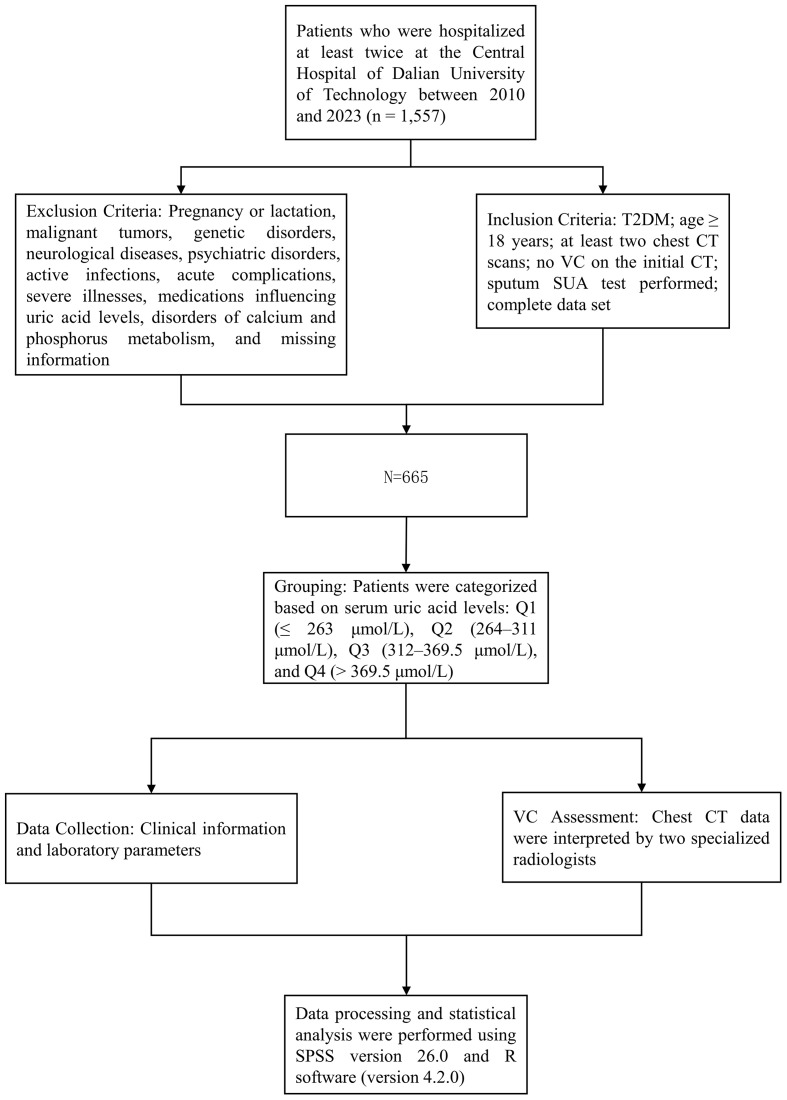
Flow chart of the study population.

A total of 665 patients with T2DM were selected based on the established inclusion and exclusion criteria. The patients were divided into four groups based on the interquartile range of baseline SUA levels, following the methodology outlined in the study by Jun et al. (21): Q1 (≤263 μmol/L), Q2 (264–311 μmol/L), Q3 (312–369.5 μmol/L), and Q4 (>369.5 μmol/L) (**Figure 1**).

### 2.2 Clinical data collection

Patient data were extracted using the YiduCloud Electronic Medical Record (EMR) Retrieval System (YiduTech Inc., Beijing, China). The collected information included personal and medical histories, such as past medical history, drug history, smoking history, and alcohol use history; physical examination metrics including height, weight, blood pressure, body mass index (BMI), and waist circumference (WC); laboratory parameters such as fasting plasma glucose (FPG), glycated hemoglobin (HbA1c), total cholesterol (TC), triglycerides (TG), low-density lipoprotein cholesterol (LDL-C), high-density lipoprotein cholesterol (HDL-C), SUA, SCr, blood urea nitrogen (BUN), alanine aminotransferase (ALT), aspartate aminotransferase (AST), and gamma-glutamyl transferase (γ-GT); and pulmonary CT data.

### 2.3 Laboratory tests and CT assessment of VC

HbA1c was measured using an automated glycohemoglobin analyzer (HLC-723G8; Tosoh Corporation, Tokyo, Japan), based on high-performance liquid chromatography (HPLC). The lipid parameters (HDL-C, TC, LDL-C, and TG), along with ALT, AST, γ-GT, SCr, SUA, and FPG, were assayed using an automatic clinical chemistry analyzer (ADVIA 2400 Chemistry System; Siemens Healthineers, Erlangen, Germany). VC was assessed using pulmonary CT scans (Discovery CT750 HD; GE HealthCare, Wauwatosa, WI, USA). The images were jointly evaluated by two specialized radiologists, who diagnosed and reported the presence of large-vessel calcification (i.e., calcified plaques in the coronary arteries or aorta) observed on the CT scans.

### 2.4 Key variable definitions and study timeline

DM: Diagnosed according to the *Guideline for the Prevention and Treatment of Type 2 Diabetes Mellitus in China (2020 edition)* (22), or previously diagnosed with DM, or currently receiving hypoglycemic medication. Hypertension: Diagnosed according to the *2018 Chinese Guidelines for Prevention and Treatment of Hypertension—Report of the Revision Committee of Chinese Guidelines for Prevention and Treatment of Hypertension* (23), or previously diagnosed with hypertension, or currently receiving antihypertensive medication. VC: VC was defined as radiologically visible macrovascular calcification on the mediastinal window of pulmonary CT images, including calcified foci or plaques in the coronary arteries and/or the aortic wall. Accordingly, VC in this study comprised coronary artery calcification (CAC), aortic calcification, or both. Radiological assessment of VC: Pulmonary CT images were independently reviewed by two trained radiologists who were blinded to patients’ SUA levels and other clinical information. Interobserver agreement for the initial VC assessments was evaluated using Cohen’s kappa coefficient. Discrepant cases were jointly reviewed, and the final diagnosis was determined by consensus.

Pulmonary CT images were reviewed and confirmed by two specialized radiology attending physicians. The estimated glomerular filtration rate (eGFR) was calculated using the CKD Epidemiology Collaboration (CKD-EPI) equation based on serum creatinine (SCr) levels (24). Follow-up Start and End Points: The follow-up start point for this study is the date of the patient’s first hospitalization, with the follow-up end point defined as the occurrence of VC.

### 2.5 Statistical analysis

All statistical analyses and graphical visualizations were performed using R software version 4.2.0, SPSS version 26.0, GraphPad Prism version 10, and the online platform Zstats (https://www.zstats.cn). Continuous variables were assessed for normality and are presented as mean ± standard deviation (SD) or median (interquartile range), as appropriate. Differences across SUA quartiles were evaluated using one-way analysis of variance or the Kruskal–Wallis test for continuous variables and the chi-square test for categorical variables. *Post-hoc* pairwise comparisons were performed using the least significant difference test, Dunnett’s T3 test, or Bonferroni correction, as appropriate. Spearman’s rank correlation analysis was used to assess the association between SUA levels and incident VC. Kaplan–Meier curves were generated to compare VC-free survival across SUA quartiles, and differences were assessed using the log-rank test. Cox proportional hazards regression models were used to evaluate the association between SUA and incident VC, with hazard ratios (HRs) and 95% confidence intervals (CIs) reported. Multivariable models were sequentially adjusted for demographic characteristics, lifestyle factors, comorbidities, metabolic parameters, medication use, renal function, and mineral metabolism markers. Linear trends across SUA quartiles were assessed by assigning the median value of each quartile and modeling it as a continuous variable. The proportional hazards assumption was evaluated using Schoenfeld residuals, and no evidence of violation was detected. Restricted cubic spline (RCS) analysis was performed to examine the dose–response association between SUA and incident VC, with P values reported for the overall association and nonlinearity. Time-dependent receiver operating characteristic (ROC) curves and Brier scores were estimated using inverse probability of censoring weighting (IPCW) at 1, 3, 5, and 10 years to assess model discrimination and prediction error while accounting for right-censored survival data. The base model was defined as the fully adjusted Model 4 without SUA, whereas the extended model additionally included SUA. ΔArea under the curve (AUC) and ΔBrier were calculated as the extended model minus the base model. The incremental predictive value of adding SUA was further assessed using integrated discrimination improvement (IDI) and category-free net reclassification improvement (NRI), with 95% confidence intervals and P values estimated by bootstrap resampling. Subgroup analyses were conducted according to age, sex, BMI, smoking status, hypertension, and eGFR, and interaction terms were tested to assess effect modification. Sensitivity analyses were conducted by excluding VC events occurring within the first year of follow-up, using an SUA cutoff of 300 μmol/L, and restricting the analysis to participants with preserved renal function, defined as eGFR ≥90 mL/min/1.73 m². All tests were two-sided, and P <0.05 was considered statistically significant.

## 3 Results

### 3.1 Comparison of clinical characteristics and biochemical indicators across groups

Patients were stratified into four quartiles (Q1–Q4) based on SUA levels: Q1 (≤263 μmol/L), Q2 (264–311 μmol/L), Q3 (312–369.5 μmol/L), and Q4 (>369.5 μmol/L).

Univariate analysis of variance, the Kruskal-Wallis rank sum test, and the chi-square test revealed no statistically significant differences (P > 0.05) among the four patient groups with respect to gender, age, alcohol use, family history, insulin therapy, lipid-lowering therapy, hypertension, pulse pressure, pulse rate, hemoglobin, HDL-C, BUN, magnesium (Mg^2+^), diastolic blood pressure, systolic blood pressure, eGFR, 2-hour postprandial glucose (2hPG), and waist-to-hip ratio. However, statistically significant differences were found in smoking status, alkaline phosphatase (ALP), TG, SCr, calcium (Ca^2+^), FPG and BMI. (P < 0.05) (**Table 1**).

**Table 1:** Intergroup comparison of patient demographics and biochemical indicators.

Variables	Q1 (*n* = 143)	Q2 (*n* = 191)	Q3 (*n* = 171)	Q4 (*n* = 160)	*P value*
Male (%)	72 (50.3)	89 (46.6)	78 (45.6)	63 (39.4)	0.276
Age (years)	59.03 ± 11.64	56.59 ± 11.37	57.19 ± 11.21	58.06 ± 11.29	0.236
Alcohol use (%)	31 (21.7)	48 (25.1)	50 (29.2)	54 (33.8)	0.095
Smoking (%)	46 (32.2)	59 (30.9)	62 (36.3)	73 (45.6)^b^	0.023
Family history (%)	60 (42.0)	82 (42.9)	75 (43.9)	65 (40.6)	0.942
Insulin therapy (%)	2 (1.4)	7 (3.7)	6 (3.5)	6 (3.8)	0.530
Lipid-lowering therapy (%)	4 (2.8)	3 (1.6)	5 (2.9)	0 (0)	0.055
Hypertension (%)	16 (11.2)	20 (10.5)	18 (10.5)	17 (10.6)	0.997
Pulse pressure (mmHg)	53.03 ± 17.25	53.66 ± 8.99	52.52 ± 13.64	53.01 ± 15.83	0.894
Pulse rate (beats/min)	77.78 ± 8.28	78.29 ± 7.39	78.12 ± 7.56	78.36 ± 7.48	0.917
Hemoglobin (g/L)	137.92 ± 14.55	139.20 ± 17.49	141.49 ± 16.57	141.29 ± 15.22	0.150
ALP (U/L)	84.65 ± 29.42	75.93 ± 20.36	77.26 ± 25.29	78.11 ± 22.81	0.009
TG (mmol/L)	1.73 ± 1.34	2.09 ± 1.94	2.06 ± 1.52	2.44 ± 2.07	0.006
HDL-C (mmol/L)	1.06 ± 0.27	1.06 ± 0.24	1.01 ± 0.22	1.05 ± 0.20	0.180
SCr (umol/L)	59.44 ± 14.56	62.88 ± 17.23	63.30 ± 14.63	69.61 ± 16.44	<0.001
BUN (mmol/L)	5.68 ± 1.86	5.65 ± 1.58	5.63 ± 1.39	5.88 ± 1.53	0.455
Mg^2+^ (mmol/L)	0.90 ± 0.07	0.89 ± 0.08	0.89 ± 0.07	0.88 ± 0.07	0.312
Ca^2+^ (mmol/L)	2.32 ± 0.11	2.34 ± 0.11	2.35 ± 0.10	2.35 ± 0.10	0.037
Fasting plasma glucose (mmol/L)	9.31 ± 2.59	8.89 ± 2.35	9.91 ± 4.03	10.59 ± 3.72	<0.001
Diastolic BP (mmHg)	83.24 ± 10.10	84.05 ± 10.65	84.46 ± 11.01	86.13 ± 12.86	0.140
Systolic BP (mmHg)	138.23 ± 22.55	139.30 ± 18.37	137.44 ± 18.77	138.11 ± 19.75	0.844
eGFR (mL/min per 1.73 m^2^)	93.36 ± 8.80	94.53 ± 8.94	94.31 ± 7.91	93.64 ± 8.67	0.568
BMI (kg/m^2^)	26.02 ± 3.77	25.51 ± 3.84	26.36 ± 3.36	26.61 ± 3.55	0.028
2-h plasma glucose (mmol/L)	16.41 ± 3.65	16.09 ± 3.48	16.42 ± 3.77	16.14 ± 3.42	0.753
Waist-to-hip ratio	0.94 ± 0.05	0.95 ± 0.06	0.94 ± 0.06	0.93 ± 0.06	0.536

Data are presented as mean ± standard deviation (SD) or n (%).TG, triglycerides; HDL-C, high-density lipoprotein cholesterol; SCr, serum creatinine; BUN, blood urea nitrogen; Mg^2+^, serum magnesium; Ca^2+^, total serum calcium; eGFR, estimated glomerular filtration rate; BP, blood pressure; BMI, body mass index.

### 3.2 Endpoint outcomes

#### 3.2.1 Overall endpoint

The two radiologists demonstrated almost perfect interobserver agreement in CT-based VC assessment, with a Cohen’s kappa coefficient of 0.822 (95% CI, 0.774–0.865). The median follow-up duration for all patients was 2.25 years. As of the follow-up cutoff date, vascular calcification had developed in 315 patients (47.4%).

#### 3.2.2 Incidence of VC at different SUA levels

**Figure 2:**
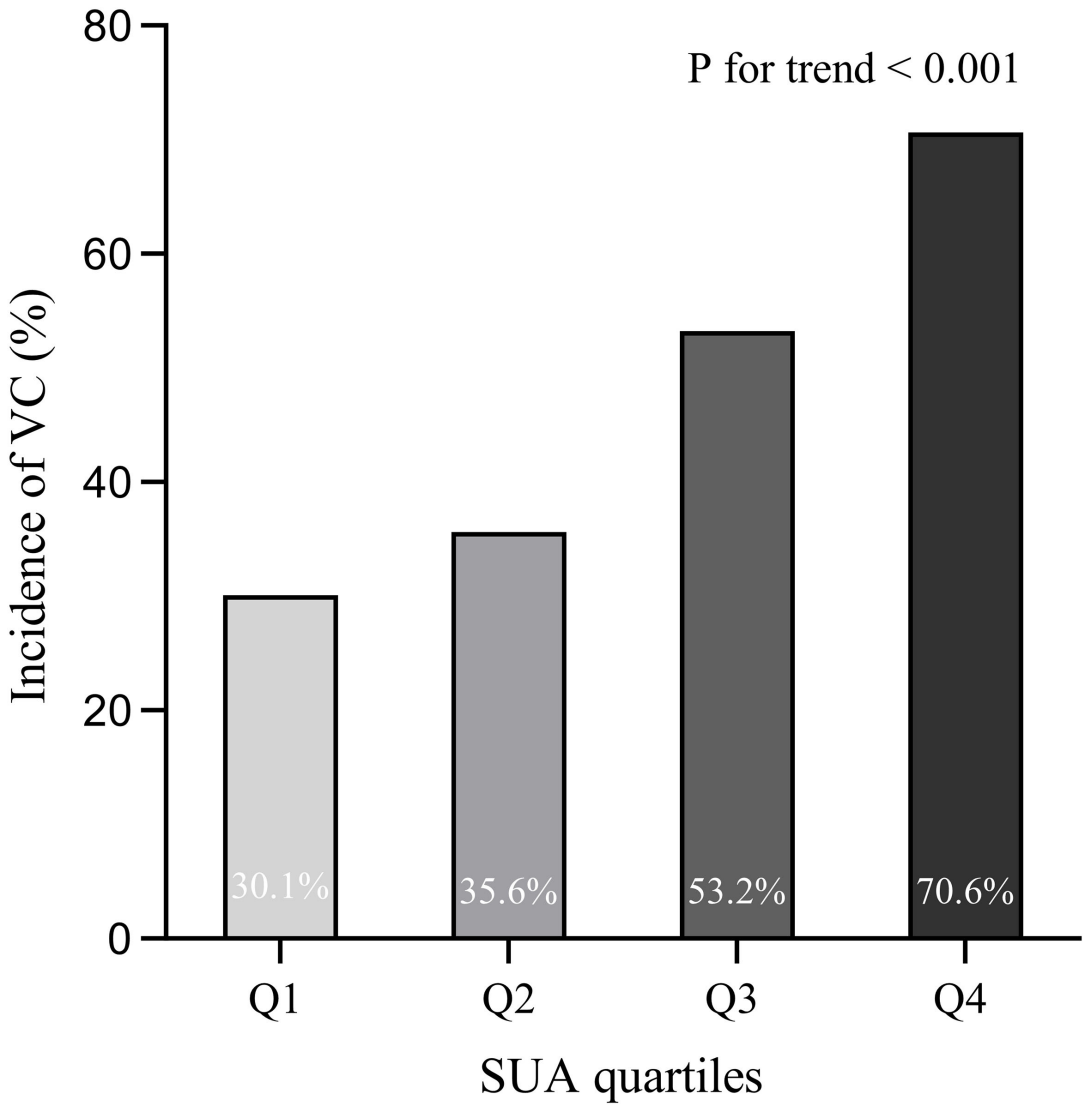
Incidence of VC across SUA quartiles.

Chi-square tests comparing the incidence of VC across four groups showed that the incidence increased with higher serum uric acid levels. The Q4 group demonstrated a higher incidence than the Q3 group, and both had higher incidences than the Q2 and Q1 groups (P < 0.05) (**Figure 2**).

### 3.3 Comparison of time to VC onset across different SUA levels

**Figure 3:**
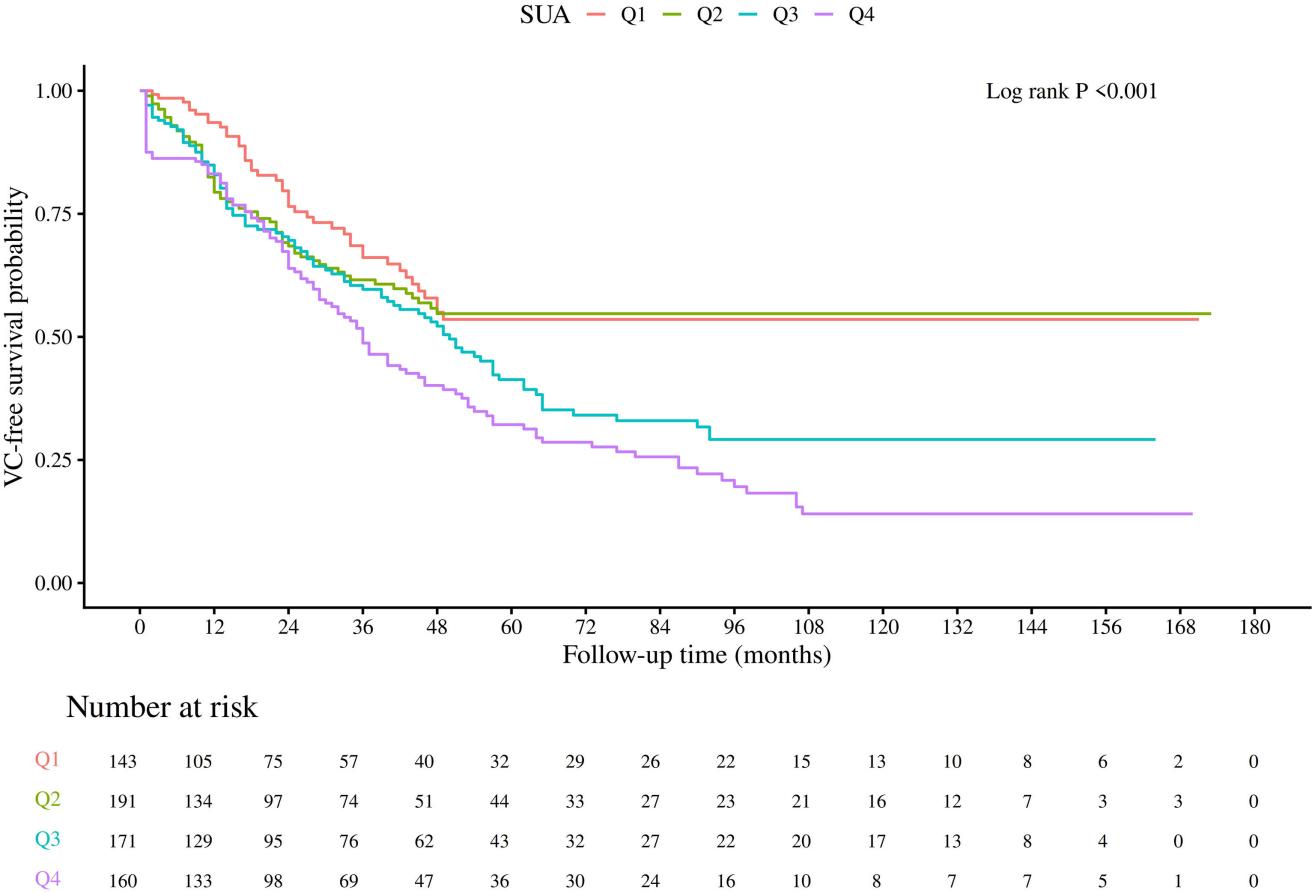
Kaplan–Meier curves for time to incident VC by SUA quartiles.

Kaplan-Meier analysis with the Log Rank (Mantel-Cox) test showed that higher SUA levels were associated with a significantly shorter time to VC (P < 0.05). Compared to the Q1 group, the time to calcification in the Q2 group was not significantly different (P > 0.05), whereas it was significantly shorter in both the Q3 and Q4 groups (P < 0.05) (**Figure 3**).

The curves depict the proportion of participants free of VC over time, with each line representing a specific SUA quartile.

### 3.4 Correlation analysis between SUA and VC

Spearman’s rank correlation analysis revealed a significant positive correlation between SUA levels and the occurrence of VC (rs = 0.306, P < 0.001).

### 3.5 Association between SUA and incident VC

Cox proportional hazards regression models were employed to evaluate the association between SUA and incident VC in patients with T2DM. As shown in **Table 2**, higher SUA levels, particularly when modeled continuously or in the upper quartiles, were associated with an increased risk of incident VC. When analyzed as a continuous variable, each 1–standard deviation (SD) increase in SUA was significantly associated with incident VC in the unadjusted model (HR 1.284, 95% CI 1.163–1.418; P < 0.001).

**Table 2:** Associations of SUA with the risk of incident VC.

SUA	Model 1		Model 2		Model 3		Model 4	
	HR (95% CI)	*P* value	HR (95% CI)	*P* value	HR (95% CI)	*P* value	HR (95% CI)	*P* value
Per 1-SD increase	1.284 (1.163–1.418)	<0.001	1.279 (1.157–1.412)	<0.001	1.258 (1.132–1.397)	<0.001	1.271 (1.138–1.420)	<0.001
Quartile 1	Ref		Ref		Ref		Ref	
Quartile 2	1.226 (0.837–1.797)	0.295	1.302 (0.887–1.910)	0.178	1.306 (0.888–1.920)	0.175	1.316 (0.891–1.944)	0.167
Quartile 3	1.690 (1.176–2.430)	0.005	1.766 (1.227–2.540)	0.002	1.726 (1.196–2.492)	0.004	1.634 (1.125–2.375)	0.010
Quartile 4	2.212 (1.557–3.144)	<0.001	2.189 (1.540–3.113)	<0.001	2.101 (1.466–3.012)	<0.001	2.075 (1.432–3.006)	<0.001
*P* for trend		<0.001		<0.001		<0.001		<0.001

Model 1 was unadjusted. Model 2 was adjusted for age, sex, alcohol use, smoking status, and family history. Model 3 was further adjusted for hypertension, body mass index (BMI), pulse pressure, triglycerides (TG), high-density lipoprotein cholesterol (HDL-C), fasting plasma glucose (FPG), insulin therapy, and lipid-lowering therapy. Model 4 was additionally adjusted for estimated glomerular filtration rate (eGFR), hemoglobin, alkaline phosphatase (ALP), serum magnesium, and serum calcium. SUA, serum uric acid; VC, vascular calcification; HR, hazard ratio; CI, confidence interval; SD, standard deviation.

This association remained significant after sequential adjustment for demographic characteristics, lifestyle factors, comorbidities, metabolic parameters, medication use, renal function, and markers of mineral metabolism. In the fully adjusted model (Model 4), each 1–SD increase in serum uric acid was associated with a 27.1% higher risk of incident vascular calcification (HR 1.271, 95% CI 1.138–1.420; P < 0.001). When SUA was categorized into quartiles, the risk of incident VC increased with higher SUA levels. Compared with the lowest quartile (Q1), the second quartile (Q2) was not significantly associated with incident VC in the fully adjusted model (HR 1.316, 95% CI 0.891–1.944; P = 0.167), whereas the third (Q3) and highest quartiles (Q4) were associated with significantly higher risks (HR 1.634, 95% CI 1.125–2.375; P = 0.010 and HR 2.075, 95% CI 1.432–3.006; P < 0.001, respectively). A significant dose–response trend was observed across SUA quartiles in all models (all P for trend < 0.001). In addition, no evidence of violation of the proportional hazards assumption was observed in models with SUA modeled either continuously or by quartiles, as indicated by nonsignificant global Schoenfeld tests (P = 0.580 and P = 0.099, respectively; **Supplementary Figures 1, 2**).

### 3.6 Dose–response association between SUA and incident VC

**Figure 4:**
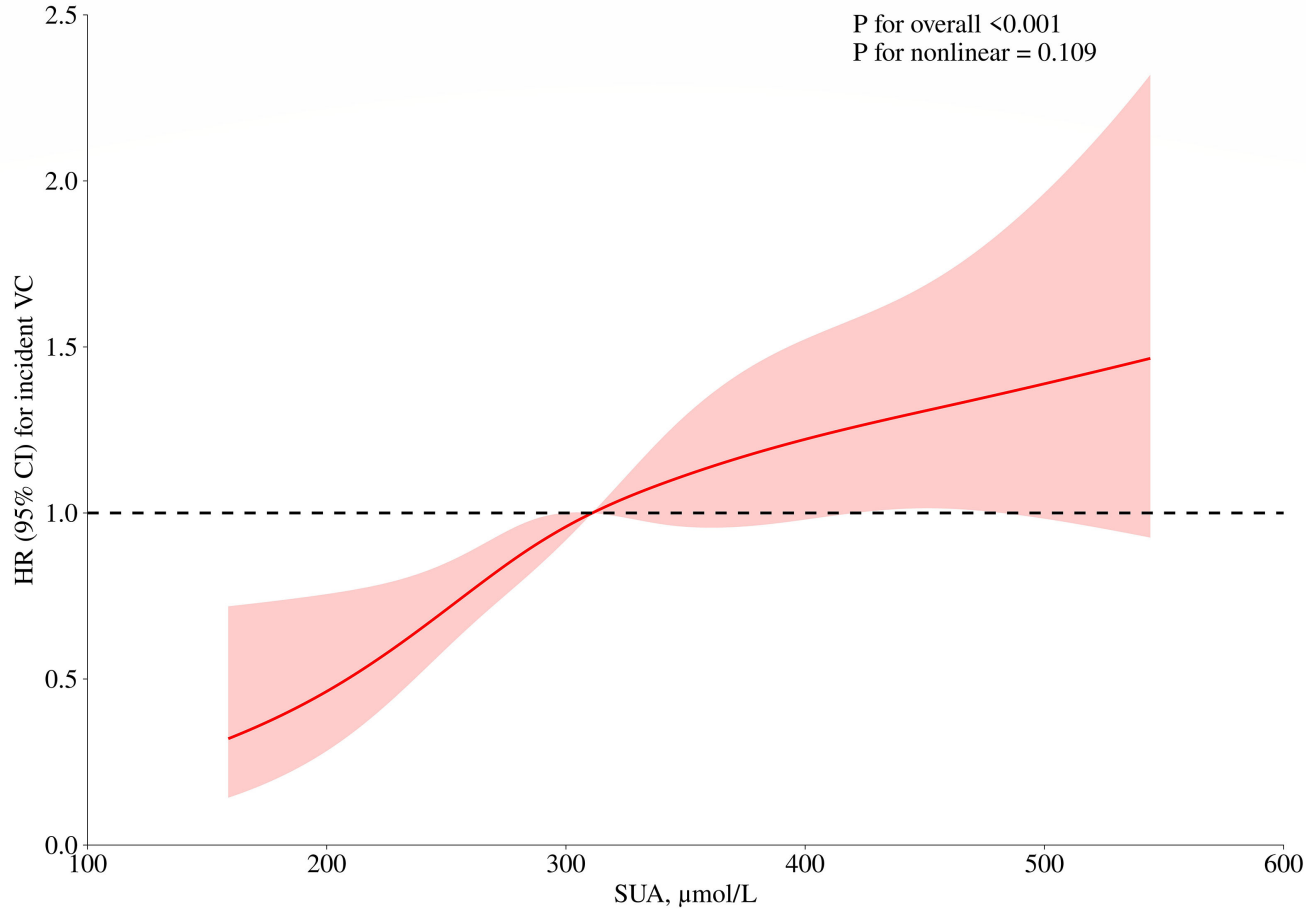
Dose–response association between SUA and incident VC.

RCS analysis was performed to further evaluate the dose–response association between SUA and incident VC after multivariable adjustment (**Figure 4**). The analysis showed a significant overall association between SUA and the risk of incident VC (P for overall association < 0.001). However, no significant evidence of nonlinearity was observed (P for nonlinearity = 0.109), suggesting an approximately linear association. As SUA levels increased, the hazard ratio for incident VC gradually increased, indicating that higher SUA levels were associated with a progressively greater risk of VC.

RCS analysis showing the dose–response association between SUA and the risk of incident VC in patients with T2DM.

### 3.7 Time-dependent predictive performance of SUA for incident VC

**Figure 5:**
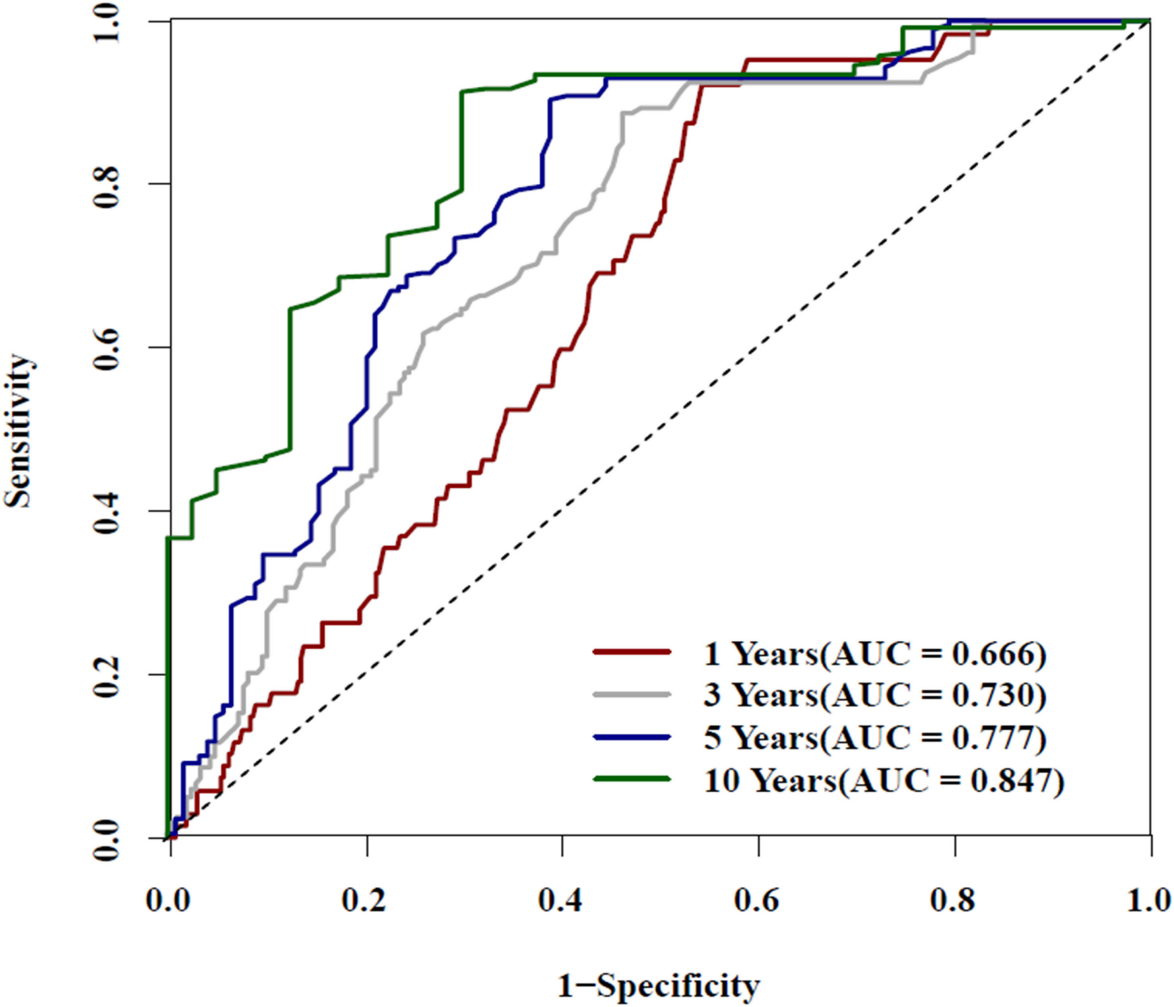
Time-dependent ROC curves for SUA in predicting incident VC.

To evaluate the predictive performance of SUA for incident VC in patients with T2DM, time-dependent ROC analysis was performed at 1, 3, 5, and 10 years using IPCW to account for right-censored observations (**Figure 5**). The time-dependent AUCs for SUA were 0.666 (95% CI, 0.607–0.724), 0.730 (95% CI, 0.678–0.781), 0.777 (95% CI, 0.723–0.832), and 0.847 (95% CI, 0.784–0.910) at 1, 3, 5, and 10 years, respectively. The corresponding numbers at risk were 501, 276, 155, and 54, respectively; therefore, the 10-year AUC should be interpreted cautiously.

After adding SUA to the fully adjusted base model, the extended model showed modest improvements in predictive performance, as reflected by numerically higher AUCs and lower Brier scores at all time points. IDI was significant at 5 and 10 years, whereas category-free NRI was not significant at any prespecified time point, suggesting modest incremental predictive value of SUA beyond conventional covariates (**Supplementary Tables 1, 2**).

Time-dependent ROC curve analysis was used to evaluate the predictive performance of SUA for incident VC in patients with T2DM.

### 3.8 Subgroup analyses

**Figure 6:**
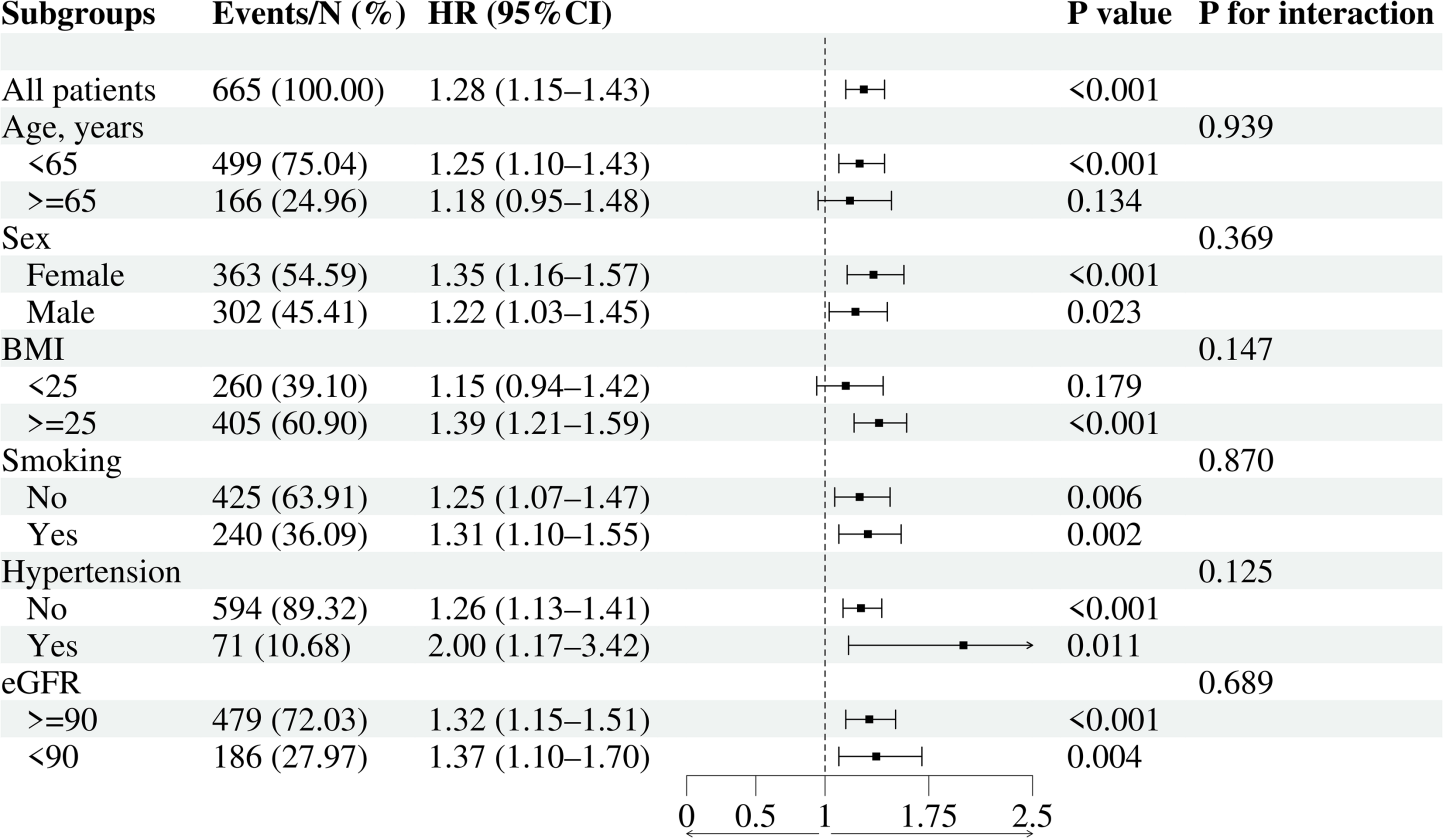
Subgroup analyses of the association between SUA and incident VC.

Subgroup analyses were conducted to evaluate the consistency of the association between SUA and incident VC across clinically relevant strata (**Figure 6**). The positive association between SUA and incident VC was generally consistent across subgroups defined by age, sex, BMI, smoking status, hypertension, and eGFR. Significant associations were observed in participants aged <65 years, in both sexes, in individuals with BMI ≥25 kg/m², among smokers and non-smokers, in participants with and without hypertension, and in those with eGFR ≥90 or <90 mL/min/1.73 m². Although associations did not reach statistical significance in participants aged ≥65 years or with BMI <25 kg/m², the effect estimates remained directionally consistent. No significant interactions were detected across any subgroup (all P for interaction >0.05), indicating that the relationship between SUA and incident VC was broadly stable across different patient groups.

Subgroup analyses were conducted according to age, sex, BMI, smoking status, hypertension, and eGFR.

### 3.9 Sensitivity analysis

The robustness of the primary findings was supported by consistent results across several sensitivity analyses. After excluding VC events that occurred within the first year of follow-up, SUA remained significantly associated with incident VC in the fully adjusted model, both when analyzed as a continuous variable and when modeled as the highest quartile. Similar findings were observed when SUA was analyzed using a cutoff value of 300 μmol/L, with SUA ≥300 μmol/L associated with an increased risk of incident VC. Furthermore, the positive association between SUA and incident VC persisted among participants with preserved renal function, defined as an eGFR ≥90 mL/min/1.73 m². Collectively, these findings support the robustness of the main results (**Supplementary Tables 3**-**5**).

## 4 Discussion

VC is classified into intimal and medial calcification. Intimal calcification occurs in the inner layer of arteries, while medial calcification refers to the deposition of calcium among smooth muscle cells within the medial layer of the arterial wall (25). Intimal calcification is a hallmark of atherosclerosis and can contribute to obstructive coronary artery disease, including angina and myocardial infarction (26). Medial calcification, characterized by the transdifferentiation of VSMCs into an osteoblastic phenotype, resembling bone formation, is commonly observed in conditions such as DM and CKD, and is closely associated with metabolic syndrome. This process leads to arterial stiffness, and both arterial stiffness and medial calcification independently predict cardiovascular mortality risk (27–33). Clinically, intimal and medial calcification frequently co-occur and may synergistically accelerate disease progression (34, 35). Overall, VC is not only a marker of early, preclinical arterial disease but also a strong predictor of future cardiovascular and cerebrovascular events. Moreover, VC is independently associated with cardiovascular mortality, even after accounting for the presence of atherosclerosis (30, 36, 37). A multicohort study reported that the incidence of intimal calcification was 37% among individuals with T2DM, compared with 17% among those with normoglycemia (38). In addition, a controlled study found that the prevalence of vascular medial calcification was 21.2% in patients with diabetes versus 5.0% in healthy controls (39).

Studies of coronary artery and aortic calcification in populations with DM similarly report higher incidence rates than those observed in the general population. In a cross-sectional study of 139 patients with DM, the prevalence of coronary artery calcification was 25.9%, compared with 7.2% in randomly selected non-diabetic controls and 14.4% in matched non-diabetic controls (40). A prospective multicohort study involving 13,376 patients revealed coronary artery calcification rates of 17%, 22%, and 37% among individuals with normal blood glucose, prediabetes, and DM, respectively (38). Furthermore, a cross-sectional study of 1,739 diabetic patients demonstrated thoracic aortic calcification in 54% of participants (41). In contrast to diabetic patients, a cross-sectional study involving 1,033 participants found that the overall prevalence of VC in the general population was 24% (42). Furthermore, another cross-sectional study of 19,725 individuals revealed that the prevalence of CAC in the general population was 21%, with a prevalence of 25% in men and 17% in women (43).

Several factors—including elevated LDL-C, insulin resistance, increased HbA1c, kidney injury markers such as the urinary albumin-to-creatinine ratio (UACR), systemic atherosclerotic burden, and disordered phosphorus metabolism—are closely associated with VC in patients with T2DM (44–47). The higher incidence of VC in diabetic populations underscores the need to clarify the causal contribution and relative importance of these factors and to identify additional, potentially novel determinants.

UA is the end product of purine metabolism. Under physiological conditions, UA production and excretion are balanced. Disruption of this homeostasis results in HUA (48). The pathological consequences of hyperuricemia extend beyond local tissue injury caused by urate crystal deposition (49). For instance, SUA has been extensively evaluated and demonstrated to be an independent predictor not only of cardiovascular mortality but also of myocardial infarction, stroke, and heart failure (50).

The relationship between VC and SUA remains unclear. Evidence is inconsistent regarding whether SUA contributes to the development and progression of VC, underscoring the need for further investigation. In a study by Jun et al. including 4,461 participants without a history of cardiovascular disease and with no or only mild coronary artery calcification at baseline, SUA was an independent predictor of progression to moderate coronary artery calcification (21). Li et al. analyzed 2,765 adults from the general population in the National Health and Nutrition Examination Survey (NHANES) database and reported a positive dose–response association between SUA levels and abdominal aortic calcification (AAC) scores (51). However, in a separate NHANES analysis of 3,016 participants, Liu et al. observed a U-shaped relationship between SUA levels and AAC risk, with calcification risk initially decreasing and then increasing as SUA rose (52). Additionally, in a study of 2,412 individuals from the general population, Neogi et al. found no association between SUA levels and CAC. The association between SUA levels and VC in patients with DM also remains unclear (20). A study of 2,731 individuals from the general population in the NHANES database suggests that DM may partially mediate the association between the uric acid–to–high-density lipoprotein cholesterol ratio (UHR) and AAC scores, as well as the association between UHR and severe abdominal aortic calcification (SAAC) (53).

Therefore, this study conducted a follow-up investigation of hospitalized patients with T2DM at the Central Hospital of Dalian University of Technology between January 2010 and January 2023. The objectives were to examine the association between SUA levels and the occurrence of VC in patients with T2DM, determine whether SUA is an independent risk factor for VC in this population, and assess the predictive value of SUA for the development of macrovascular calcification, including coronary artery and aortic calcification.

CAC is most commonly assessed using electrocardiographic (ECG)-gated cardiac electron beam CT or multidetector CT (MDCT). The extent of calcification is quantified using the coronary artery calcification score, also known as the Agatston score (54). In contrast, a standardized system for assessing aortic calcification has not yet been fully established (55). Currently, available evaluation methods include CT, lateral dual-energy X-ray absorptiometry (DXA), and chest radiography, among others (56, 57). The aforementioned methods each have distinct advantages and disadvantages regarding resolution, accessibility, and cost. This study employed routine lung CT examinations to assess mediastinal window calcification of the great vessels. Compared to the methods mentioned above, this approach offers several advantages: it utilizes routinely acquired clinical images, eliminating the need for additional scans or radiation exposure. In addition to evaluating arterial calcification, it simultaneously allows for the observation of pulmonary and cardiovascular structures. This method demonstrates higher clinical feasibility and comprehensive diagnostic value, making it well-suited for low-cost screening in real-world populations.

The results of this study indicate that the incidence of VC in patients with T2DM is 47.4%, which is higher than the 25.9% and 37% rates of CAC reported in previous studies (38, 40), and comparable to the 54% incidence of AAC reported in other studies (41). It also exceeds the 24% VC rate and the 21% CAC rate observed in the general population in prior studies (42, 43). Additionally, this study found that the risk of VC increased with rising SUA levels in patients with T2DM. The incidence of VC in the Q4 group was significantly higher than in the other three groups (70.6% vs. 53.2% vs. 35.6% vs. 30.1%) (P<0.05).

Additionally, this study found that serum uric acid levels were positively correlated with the occurrence of large vessel calcification (coronary artery and aortic calcification) (rs = 0.306, P < 0.001). This finding aligns with a cross-sectional study of middle-aged and elderly women, which assessed aortic arch calcification via radiography and demonstrated an independent association between serum uric acid and aortic arch calcification. Among all 5,920 participants, calcification was detected in 851 individuals (14.4%). For all participants, the prevalence of aortic arch calcification exhibited an increasing trend across ascending SUA tertiles, with 12.31%, 13.53%, and 17.42% in the T1, T2, and T3 groups, respectively (37). Another study based on an asymptomatic population used low-dose CT to assess CAC and found a positive correlation between SUA levels and the total CAC score (r = 0.281, P < 0.001). Within the SUA tertile groups, the median total calcium score and the frequency of CAC > 0 and CAC > 300 significantly increased across the three groups as SUA levels rose (58). In contrast, a Brazilian study involving a healthy elderly population, which employed coronary CT to assess CAC, found no association between elevated SUA levels and CAC in individuals aged over 80 years. Across increasing tertiles of SUA, no significant differences were observed in either the presence of CAC or CAC burden (CAC > 0 or CAC > 400) (P = 0.988) (59). All the aforementioned studies were conducted in non-diabetic populations and employed varying methodologies for assessing VC, which may explain their divergent conclusions. The present study is the first to investigate the association between SUA and VC specifically in a diabetic population. It enrolled Chinese patients with T2DM, who may also have comorbid conditions such as hypertension. VC status was defined using pulmonary CT.

After multivariable adjustment, SUA remained independently associated with incident macrovascular calcification in patients with T2DM. This finding aligns with the results of Yan et al.’s cross-sectional study in a middle-aged and elderly general population (37). The study enrolled 5,920 participants aged over 45 years with no baseline cardiovascular disease. In Multivariate Model 1 (adjusted for age, sex, hypertension, and eGFR) and Multivariate Model 2 (further adjusted for BMI, dyslipidemia, and DM), multivariate logistic regression analysis demonstrated that SUA levels were independently associated with aortic arch calcification in all participants. Compared with the lowest tertile of SUA, the odds ratios (ORs) for the highest tertile were 1.37 (95% CI, 1.13–1.66) and 1.41 (95% CI, 1.16–1.72) (P < 0.001). This study focused on patients with T2DM, employing Cox regression analysis for survival data, whereas Yan et al.’s research encompassed the general middle-aged and elderly population, utilizing logistic regression on cross-sectional data. Despite differences in study populations and analytical methods, both studies support an independent association between SUA and VC. The convergence of findings across distinct populations and methodologies strengthens the evidence supporting SUA’s potential pivotal role in the pathophysiology of VC, suggesting a degree of universality in this association.

In this study, several baseline characteristics differed across SUA quartiles, including smoking status, ALP, TG, SCr, serum calcium, FPG, and BMI. Current evidence suggests that hyperuricemia is not only associated with gout but may also be closely related to hypertension, DM, metabolic syndrome, CKD, hypertriglyceridemia, obesity, atherosclerotic heart disease, and other cardiovascular diseases (60, 61). Furthermore, in patients with T2DM, multiple interrelated mechanisms, including endothelial dysfunction and dysregulated lipid metabolism, may jointly contribute to the progression of VC (7). Thus, the increased VC risk observed among patients with higher SUA levels may reflect not only the potential effect of SUA itself but also, at least in part, the influence of the aforementioned confounding factors. Previous studies have suggested that ALP, TG, SCr, serum calcium, FPG, and BMI are associated with the onset and progression of VC or CAC, potentially through multiple pathways, including oxidative stress, inflammation, dysregulated glucose and lipid metabolism, insulin resistance, renal dysfunction, and disturbances in mineral metabolism (7, 62–67). Accordingly, these imbalanced variables, or their clinically more appropriate surrogates, were included in the stepwise multivariable Cox regression models, including smoking status, BMI, TG, FPG, eGFR, ALP, and serum calcium. After adjustment for these covariates, the association between SUA and incident VC remained statistically significant, suggesting that this relationship was not fully explained by the observed baseline differences.

Current basic research evidence supports an association between HUA and VC. VC refers to the process of calcium phosphate crystal formation within the vascular wall, mediated by VSMCs, although its molecular mechanisms remain unclear (68). Liu et al. demonstrated through animal models that the hyperuricemic group exhibited earlier onset and more severe progression of aortic calcification compared to both the normal diet and high-fat diet groups. Furthermore, the duration of hyperuricemic exposure was correlated with increased severity of calcium deposition in the vascular media (69). On the other hand, Yan et al. further demonstrated through animal and cellular models that HUA inhibits osteoblast differentiation and proliferation both *in vivo* and *in vitro*, while simultaneously promoting osteoblast-like transdifferentiation of VSMCs, thereby increasing the risk of VC. Specifically, compared to the control group, VSMCs from HUA rat specimens exhibited significantly elevated calcium content, suggesting that HUA promotes VC *in vivo*. Furthermore, in VSMCs isolated from HUA rats, the expression of Runx2, Sp7, Bglap, Col1a1, SM22a, and Acta2 was markedly upregulated, confirming the role of SUA in promoting VC (17).

Time-dependent ROC analysis showed that SUA had AUCs of 0.666, 0.730, 0.777, and 0.847 at 1, 3, 5, and 10 years, respectively. Adding SUA to the fully adjusted base model modestly improved predictive performance, with higher AUCs and lower Brier scores at all prespecified time points. IDI was significant at 5 and 10 years, whereas category-free NRI was not, suggesting improved average discrimination but limited reclassification gain beyond conventional covariates. The data-derived cutoffs clustered around 300 μmol/L, a value substantially lower than the 420 μmol/L target recommended in current guidelines (70). However, this threshold should be interpreted as exploratory and hypothesis-generating rather than as a validated treatment target. Because urate-lowering therapy was not evaluated in this observational study, these findings do not establish that maintaining SUA below 300 μmol/L would prevent or delay VC. Instead, SUA may serve as an accessible biomarker for VC risk stratification in patients with T2DM, pending prospective external validation.

This study has several limitations. First, VC was assessed using routine pulmonary CT images obtained in clinical practice, rather than dedicated ECG-gated cardiac CT, Agatston scoring, standardized aortic calcification scoring, or intravascular imaging. Accordingly, the outcome represents radiologically visible macrovascular calcification on mediastinal-window pulmonary CT, which may have limited sensitivity for mild or early lesions and does not quantify calcification burden, lesion location, or progression. Although interobserver agreement was almost perfect, this finding supports reproducibility rather than validity against reference-standard methods. In addition, the composite CAC/aortic calcification outcome precluded separate evaluation of coronary and aortic calcification, which may differ in pathophysiology and clinical implications. Second, this retrospective, hospital-based cohort required at least two hospitalizations and repeated pulmonary CT examinations, which may have introduced selection, surveillance, survivor, and immortal-time biases. Included patients may have been sicker or more closely monitored than the broader T2DM population; VC events occurring outside the study hospital may have been missed; and the exact timing of VC onset between CT scans could not be determined. These factors limit the generalizability of our findings to all patients with T2DM. Third, although the analyses adjusted for demographic, lifestyle, metabolic, renal, medication-related, and mineral metabolism factors, residual confounding remains possible. Data on diabetes duration, HbA1c trajectory, albuminuria, serum phosphate, vitamin D, parathyroid hormone (PTH), fibroblast growth factor 23 (FGF-23), high-sensitivity C-reactive protein (hs-CRP), statin intensity, antihypertensive therapy, antiplatelet therapy, bisphosphonate use, and other cardiovascular medications were unavailable or incompletely captured.

Fourth, the predictive findings should be interpreted with caution. Although IPCW-based time-dependent ROC analyses were used to account for right censoring, the median follow-up was 2.25 years, and only 54 participants remained at risk at 10 years; therefore, the 10-year AUC was less stable. The approximately 300 μmol/L SUA threshold was data-derived and unvalidated; therefore, it should not be interpreted as a clinical treatment target or as evidence that lowering SUA prevents VC. Prospective multicenter studies using standardized CT-based calcification assessment, as well as interventional studies of urate-lowering strategies, are needed to confirm the clinical utility of SUA for VC risk stratification and to determine whether SUA modification can reduce VC progression.

## 5 Conclusion

In this retrospective cohort of patients with T2DM, higher baseline SUA levels were independently associated with an increased risk of incident radiologically visible macrovascular calcification, including CAC, aortic calcification, or both. SUA provided modest incremental predictive value beyond conventional clinical covariates and may help identify patients with T2DM who are at higher risk of developing VC. The data-derived threshold of approximately 300 μmol/L should be regarded as exploratory and hypothesis-generating rather than as a validated treatment target. Because no urate-lowering intervention was tested, these findings do not establish whether lowering SUA prevents VC. Further prospective multicenter studies using standardized calcification assessment, as well as interventional studies of urate-lowering strategies, are needed to validate the risk-stratification value of SUA and to determine whether SUA reduction can modify VC risk.

## Data Availability

The raw data supporting the conclusions of this article will be made available by the authors, without undue reservation.
