# Supplementary material for: Association of serum uric acid with incident vascular calcification in patients with type 2 diabetes mellitus: a retrospective cohort study
Source: Front Endocrinol (Lausanne). 2026 Jul 28;17:1893598. doi: 10.3389/fendo.2026.1893598 (PMC13459047; doi:10.3389/fendo.2026.1893598)
Supplement: Supplementary file 1 [file Supplementaryfile1.docx]

**Supplemental files**

**Association of Serum Uric Acid With Incident Vascular Calcification in Patients With Type 2 Diabetes Mellitus: A Retrospective Cohort Study**

^1^Department of Endocrinology, Central Hospital of Dalian University of Technology, 826 Xinnan Road, Shahekou District, Dalian City, Liaoning Province, 116033, China.

**^2^**Graduate School, Dalian Medical University, Dalian, China.

^3^Laboratory Pathology Department, Ningbo Clinical Pathology Diagnosis Center, Ningbo, 315021, China.

^4^Department of Respiratory Medicine, Yanhu District People's Hospital of Yuncheng, Yuncheng City, 044000, China

^5^Department of Neuroendocrine Pharmacology, School of Pharmacy, China Medical University, Shenyang, 210009, China.

*Correspondence: Guosheng Li, [guoshengli998@163.com](mailto:guoshengli998@163.com); Xuhan Liu, xuhanliu281277@163.com

^†^These authors contributed equally to this work.

**Table of contents**

**Table S1** Time-dependent AUCs and Brier scores for SUA alone, the base model, and the SUA-extended model.

**Table S2** Incremental predictive value of adding SUA to the base model at prespecified time points.

**Table S3** Association between SUA and incident VC after exclusion of early events.

**Table S4** Association between SUA ≥300 μmol/L and incident VC.

**Table S5** Association between SUA and incident VC among participants with eGFR ≥90 mL/min/1.73 m².

**Figure S1** Schoenfeld residuals for testing the proportional hazards assumption in the Cox model with SUA per standard deviation increase.

**Figure S2** Schoenfeld residuals for testing the proportional hazards assumption in the Cox model with SUA quartiles.

**Table S1** Time-dependent AUCs and Brier scores for SUA alone, the base model, and the SUA-extended model.

| **Time point** | **SUA AUC (95% CI)** | **Base AUC** | **Extended AUC** | **ΔAUC** | **Base Brier** | **Extended Brier** | **ΔBrier** | **Number at risk** |
| --- | --- | --- | --- | --- | --- | --- | --- | --- |
| 1 year | 0.666 (0.607–0.724) | 0.628 | 0.642 | +0.014 | 0.1285 | 0.1279 | -0.0006 | 501 |
| 3 years | 0.730 (0.678–0.781) | 0.651 | 0.672 | +0.021 | 0.2280 | 0.2259 | -0.0021 | 276 |
| 5 years | 0.777 (0.723–0.832) | 0.638 | 0.701 | +0.063 | 0.2366 | 0.2320 | -0.0046 | 155 |
| 10 years | 0.847 (0.784–0.910) | 0.642 | 0.736 | +0.094 | 0.2151 | 0.2092 | -0.0059 | 54 |

Time-dependent AUCs and Brier scores were estimated using IPCW to account for right censoring. SUA AUC refers to the AUC of SUA alone. The base model was Model 4 without SUA, and the extended model was Model 4 with SUA. ΔAUC was calculated as Extended AUC minus Base AUC, and ΔBrier as Extended Brier minus Base Brier; negative ΔBrier values indicate lower prediction error. Number at risk indicates the number of participants remaining under observation at each time point. Model 4 was adjusted for age, sex, alcohol use, smoking status, family history, hypertension, BMI, pulse pressure, TG, HDL-C, FPG, insulin therapy, lipid-lowering therapy, eGFR, hemoglobin, ALP, serum magnesium, and serum calcium.

**Table S2** Incremental predictive value of adding SUA to the base model at prespecified time points.

| **Time point** | **IDI** | **95% CI** | ***P* value** | **Category-free NRI** | **95% CI** | ***P* value** |
| --- | --- | --- | --- | --- | --- | --- |
| 1 year | 0.004 | −0.006–0.019 | 0.382 | 0.062 | −0.059–0.183 | 0.394 |
| 3 years | 0.008 | −0.003–0.024 | 0.182 | 0.043 | −0.048–0.138 | 0.348 |
| 5 years | 0.016 | 0.001–0.038 | 0.036 | 0.088 | −0.018–0.190 | 0.118 |
| 10 years | 0.032 | 0.008–0.062 | 0.006 | 0.143 | −0.011–0.294 | 0.062 |

IDI and category-free NRI were calculated at each prespecified time point using survival-based methods. Ninety-five percent confidence intervals and P values were estimated by bootstrap resampling. The base model included the Model 4 covariates, and the extended model further included SUA. Model 4 was adjusted for age, sex, alcohol use, smoking status, family history, hypertension, BMI, pulse pressure, TG, HDL-C, FPG, insulin therapy, lipid-lowering therapy, eGFR, hemoglobin, ALP, serum magnesium, and serum calcium.

**Table S3** Association between SUA and incident VC after exclusion of early events.

| **Variable** | **Model 1** |  | **Model 2** |  | **Model 3** |  | **Model 4** |  |
| --- | --- | --- | --- | --- | --- | --- | --- | --- |
|  | **HR (95% CI)** | ***P* value** | **HR (95% CI)** | ***P* value** | **HR (95% CI)** | ***P* value** | **HR (95% CI)** | ***P* value** |
| **SUA** |  |  |  |  |  |  |  |  |
| Per 1 SD increase | 1.344 (1.195–1.512) | <0.001 | 1.341 (1.192–1.509) | <0.001 | 1.307 (1.153–1.481) | <0.001 | 1.315 (1.149–1.504) | <0.001 |
| Quartiles 1 | Ref |  | Ref |  | Ref |  | Ref |  |
| Quartiles 2 | 0.720 (0.446– 1.163) | 0.179 | 0.743 (0.459–1.203) | 0.227 | 0.758 (0.466–1.231) | 0.263 | 0.744 (0.455–1.214) | 0.237 |
| Quartiles 3 | 1.423 (0.942–2.149) | 0.094 | 1.469 (0.971–2.221) | 0.069 | 1.414 (0.931–2.149) | 0.105 | 1.269 (0.828–1.944) | 0.275 |
| Quartiles 4 | 2.034 (1.373–3.014) | <0.001 | 2.037 (1.374–3.021) | <0.001 | 1.915 (1.278–2.869) | <0.001 | 1.775 (1.167–2.699) | 0.007 |
| *P* for trend |  | <0.001 |  | <0.001 |  | <0.001 |  | <0.001 |

Model 1 was unadjusted.

Model 2 was adjusted for age, sex, alcohol use, smoking status, and family history.

Model 3 was further adjusted for hypertension, body mass index (BMI), pulse pressure, TG, HDL-C, FPG, insulin therapy, and lipid-lowering therapy.

Model 4 was additionally adjusted for eGFR, hemoglobin, ALP, serum magnesium, and serum calcium.

**Table S4** Association between SUA ≥300 μmol/L and incident VC.

| **Variable** | **Model 1** |  | **Model 2** |  | **Model 3** |  | **Model 4** |  |
| --- | --- | --- | --- | --- | --- | --- | --- | --- |
|  | **HR (95% CI)** | ***P* value** | **HR (95% CI)** | ***P* value** | **HR (95% CI)** | ***P* value** | **HR (95% CI)** | ***P* value** |
| **SUA** |  |  |  |  |  |  |  |  |
| <300 | Ref |  | Ref |  | Ref |  | Ref |  |
| ≥300 | 1.933 (1.518–2.461) | <0.001 | 1.923 (1.509–2.451) | <0.001 | 1.865 (1.453–2.395) | <0.001 | 1.796 (1.393–2.317) | <0.001 |

Model 1 was unadjusted.

Model 2 was adjusted for age, sex, alcohol use, smoking status, and family history.

Model 3 was further adjusted for hypertension, BMI, pulse pressure, TG, HDL-C, FPG, insulin therapy, and lipid-lowering therapy.

Model 4 was additionally adjusted for eGFR, hemoglobin, ALP, serum magnesium, and serum calcium.

**Table S5** Association between SUA and incident VC among participants with eGFR ≥90 mL/min/1.73 m².

| **Variable** | **Model 1** |  | **Model 2** |  | **Model 3** |  | **Model 4** |  |
| --- | --- | --- | --- | --- | --- | --- | --- | --- |
|  | **HR (95% CI)** | ***P* value** | **HR (95% CI)** | ***P* value** | **HR (95% CI)** | ***P* value** | **HR (95% CI)** | ***P* value** |
| **SUA** |  |  |  |  |  |  |  |  |
| Per 1 SD increase | 1.321 (1.170–1.492) | <0.001 | 1.359 (1.199–1.540) | <0.001 | 1.304 (1.141–1.491) | <0.001 | 1.289 (1.123–1.480) | <0.001 |
| Quartiles 1 | Ref |  | Ref |  | Ref |  | Ref |  |
| Quartiles 2 | 1.201 (0.768–1.879) | 0.422 | 1.343 (0.856–2.108) | 0.199 | 1.361 (0.864–2.145) | 0.184 | 1.314 (0.832–2.076) | 0.242 |
| Quartiles 3 | 1.597 (1.049–2.432) | 0.029 | 1.569 (1.028–2.394) | 0.037 | 1.503 (0.979–2.308) | 0.062 | 1.428 (0.921–2.213) | 0.111 |
| Quartiles 4 | 2.227 (1.476–3.360) | <0.001 | 2.382 (1.573–3.606) | <0.001 | 2.168 (1.413–3.325) | <0.001 | 2.042 (1.319–3.161) | 0.001 |
| *P* for trend |  | <0.001 |  | <0.001 |  | <0.001 |  | <0.001 |

Model 1 was unadjusted.

Model 2 was adjusted for age, sex, alcohol use, smoking status, and family history.

Model 3 was further adjusted for hypertension, BMI, pulse pressure, TG, HDL-C, FPG, insulin therapy, and lipid-lowering therapy.

Model 4 was additionally adjusted for hemoglobin, ALP, serum magnesium, and serum calcium.

**Figure S1** Schoenfeld residuals for testing the proportional hazards assumption in the Cox model with SUA per standard deviation increase.

 **Figure S2** Schoenfeld residuals for testing the proportional hazards assumption in the Cox model with SUA quartiles.
